# CMV-Responsive CD4 T Cells Have a Stable Cytotoxic Phenotype Over the First Year Post-Transplant in Patients Without Evidence of CMV Viremia

**DOI:** 10.3389/fimmu.2022.904705

**Published:** 2022-06-28

**Authors:** Lauren E. Higdon, Ayah A. Ahmad, Steven Schaffert, Kenneth B. Margulies, Jonathan S. Maltzman

**Affiliations:** ^1^Department of Medicine, Nephrology, Stanford University, Palo Alto, CA, United States; ^2^Macaulay Honors College, Hunter College, The City University of New York, New York, NY, United States; ^3^Institute for Immunity, Transplantation and Infection, Stanford University, Stanford, CA, United States; ^4^Department of Medicine/Biomedical Informatics, Stanford University, Stanford, CA, United States; ^5^Cardiovascular Institute, Perelman School of Medicine, University of Pennsylvania, Philadelphia, PA, United States; ^6^Geriatric Research Education and Clinical Center, Veteran's Affairs Palo Alto Health Care System, Palo Alto, CA, United States

**Keywords:** cytomegalovirus, CD4 T cells, transplant, T-helper 1 (Th1) cells, cytotoxic, T-cell receptor (TCR), clonal expansion

## Abstract

Cytomegalovirus (CMV) infection is a known cause of morbidity and mortality in solid organ transplant recipients. While primary infection is controlled by a healthy immune system, CMV is never eradicated due to viral latency and periodic reactivation. Transplantation and associated therapies hinder immune surveillance of CMV. CD4 T cells are an important part of control of CMV reactivation. We therefore investigated how CMV impacts differentiation, functionality, and expansion of protective CD4 T cells from recipients of heart or kidney transplant in the first year post-transplant without evidence of CMV viremia. We analyzed longitudinal peripheral blood samples by flow cytometry and targeted single cell RNA sequencing coupled to T cell receptor (TCR) sequencing. At the time of transplant, CD4 T cells from CMV seropositive transplant recipients had a higher degree of immune aging than the seronegative recipients. The phenotype of CD4 T cells was stable over time. CMV-responsive CD4 T cells in our transplant cohort included a large proportion with cytotoxic potential. We used sequence analysis of TCRαβ to identify clonal expansion and found that clonally expanded CMV-responsive CD4 T cells were of a predominantly aged cytotoxic phenotype. Overall, our analyses suggest that the CD4 response to CMV is dominated by cytotoxicity and not impacted by transplantation in the first year. Our findings indicate that CMV-responsive CD4 T cells are homeostatically stable in the first year after transplantation and identify subpopulations relevant to study the role of this CD4 T cell population in post-transplant health.

## Introduction

The herpesvirus cytomegalovirus (CMV) causes significant disease burden in transplant recipients (1). Upon immune control of primary infection, CMV establishes latency and reactivates periodically (2). While the virus is typically controlled by the immune system, transplant immunosuppression impairs immunity to CMV, resulting in uncontrolled reactivation (3). While CD8 T cells are critical for immune control of reactivation (4), CD4 T cells are also known to contribute (5–7) but have been less thoroughly studied to date. In-depth analysis of CMV-responsive CD4 T cells is necessary to fully grasp the effects of transplantation and associated therapies on immunity to CMV in seropositive individuals.

T cell immunity to CMV is altered in the context of transplantation. Studies in CMV-infected transplant recipients and infants found that lack of effective CD4 T cell responses resulted in more severe disease and prolonged viral shedding (5, 7–10). Furthermore, decreased frequencies of CMV-specific CD4 T cells were associated with development of CMV viremia in the first months after kidney transplant (8). A lack of CMV-specific CD4 T cells was also associated with higher risk of recurrent CMV viremia in hematopoietic stem cell transplant recipients (6). Thus, CD4 T cell responses are crucial to immune control of CMV after transplantation.

CMV infection has a distinctive impact on T cell differentiation. Latency and reactivation lead to memory inflation, or expansion of CMV-responsive CD8 T cells over decades (11–15). In contrast, CD4 T cells do not undergo memory inflation, though CMV-specific CD4 T cells expand during memory responses relative to the primary response (8). Upon infection, CD4 T cells recruit CD8 T cells and other effector cells to sites of viral replication. CD4 T cells also promote entry of naïve CD8 T cells and B cells to lymph nodes (16). However, CD4 T cells have an additional independent role in anti-viral immunity, namely producing interferon gamma (IFNγ) and tumor necrosis factor α (TNFα) and lysing CMV-infected cells (17–19). Understanding the distinct role of CD4 T cells is important to understanding immunity to CMV.

Antiviral CD4 T cells typically have a T-helper 1 (Th1) phenotype (20), producing IFNγ and expressing the transcription factor T-box expressed in T cells (T-bet, gene name *TBX21*). Other transcription factors further define CD4 T cell differentiation states. For instance, GATA Binding Protein 3 (GATA3) is required to induce a T-helper 2 (Th2) state, retinoid orphan receptor gamma t (RORγt, gene name *RORC*) is required to induce a Th17 state, and B-cell lymphoma 6 (BCL6) is required to induce a follicular helper T cell (Tfh) state (21). RUNX1 and RUNX3 promote thymic differentiation to CD4 or CD8 T cell states respectively (22). RUNX3 has also been shown to promote cytotoxic activity by CD4 T cells (23, 24).

To address how homeostasis of CMV-responsive CD4 T cells changes after transplantation, we assessed CD4 T cells from heart and kidney transplant recipients. The patients in the study did not have CMV viremia detected during the study course, and therefore represent a population with clinically-controlled CMV. We had previously analyzed CMV-responsive T cells by flow cytometry and by targeted single cell T cell receptor (TCR) and RNA sequencing, and focused on CD8 T cells because we observed greater phenotypic variation in CD8 T cells than CD4 T cells (25, 26). However, we did observe variation within the CD4 population as well, and therefore reanalyze the data here to address similar questions for CD4 T cells. We found that when compared to CMV seronegative (CMV^–^) recipients, CMV^+^ transplant recipients had evidence of enhanced CD4 T cell aging pre-transplant, with stable phenotypes in the first year post-transplant. We also found that clonally expanded CMV-responsive CD4 T cells were enriched for an aged cytotoxic phenotype and stably maintained this phenotype from pre- to a year post-transplant. Overall, our analyses indicate that heart or kidney transplant and associated therapies have limited impact on the CD4 T cell repertoire in CMV-seropositive individuals, but that CMV dramatically remodels the CD4 T cell repertoire independent of transplantation.

## Materials and Methods

### Human Subjects

Recipients of heart or kidney transplant were enrolled pre-transplant or within the first year after transplant at the University of Pennsylvania, Stanford University or the Veterans Administration Palo Alto Health Care System as described (25). Analysis of CD8 T cells in these cohorts by flow cytometry (25) and targeted single cell sequencing were previously described in detail (26, 27). Induction therapy varied based on transplanted organ and center (**Supplementary Tables 1, 2**). Blood samples were collected as described (25). Both recipients and organ donors were tested for CMV serostatus pre-transplant. This study was approved by the IRBs at the University of Pennsylvania (protocol number 817637) as well as the VA Palo Alto Health Care System and Stanford University (protocol number 38882). Identifiable source information was blinded to those completing studies. These studies were in accordance with the Declaration of Helsinki and all participants gave written informed consent prior study inclusion. Blood was collected and processed for isolation of peripheral blood mononuclear cells (PBMC) as described (28).

### Cell Stimulation

PBMC were thawed, rested, and stimulated with peptide libraries for the immunodominant CMV polypeptide immediate early-1 (IE-1, GenScript, Piscataway, NJ) (29) as described (11, 30). Briefly, cells were stimulated with the IE-1 library (0.8 μg/mL) at 37°C and 5% CO_2_, for five or six hours with an unstimulated control used for each sample. Antibody to human CD107a (BioLegend, San Diego, CA) was added concurrently with the peptide library. Stimulation was stopped by addition of 3 mL cold PBS (Thermo Fisher).

For samples that were subsequently stained with intracellular cytokine staining, after the first hour, brefeldin A (2 mg/mL; Life Technologies), monensin (BD Golgistop at 0.7 lL/mL) were added as described (11). After six hours stimulation cells were detached from tubes *via* incubation for 10 min in PBS with 2 mM EDTA (Thermo Fisher) at 37°C. For samples that were subsequently sorted, after five hours stimulation cells were stained using the interferon gamma (IFNγ) Secretion Assay (Miltenyi) following the manufacturer’s protocol as described (26).

### Stain for Flow Cytometric Phenotyping

Cells were stained as described (25) using antibodies conjugated to fluorescein, phycoerythrin (PE) or PE conjugates, allophycocyanin (APC) or APC conjugates, Alexa Fluor 700, Brilliant Violet dyes 421, 570, 605, 650, 711, or 785, and Brilliant Ultraviolet dyes 395, 496, and 737. The antibodies were specific for anti-human CD3 (HIT3a), CD4 (S3.5), CD8 (RPA-T8), CD14 (61D3), CD16 (3G8), CD19 (HIB19), CCR7 (G043H7), CD107a (H4A3), CD45RO (UCHL1), CD45RA (HI100), CD57(HNK1), PD-1 (EH12.2H7), T-bet (4B10), interferon (IFN)γ (B27), and tumor necrosis factor (TNF)α (mAb11) and were purchased from BioLegend, eBioscience, BD, Life Technologies, Abcam (Cambridge, UK), or Beckman-Coulter (Pasadena, CA). Cells were also stained with Zombie Aqua dye (BioLegend).

### Staining for Sorting

Cells were surface stained as described (30) with antibodies to CD4 (RPA-T4), CD8 (SK1), CD3 (OKT3), CD14 (61D3), CD16 (3G8), CD19 (HIB19), CD57 (HNK1), CD45RA (HI100), and CD27 (O323) from BioLegend and Abcam and Zombie Aqua dye (BioLegend). For sorting, cells were in PBS with 0.5% bovine serum albumin and 2 mM EDTA.

### Compensation Controls

Compensation controls were prepared by using unstained and single stained cells, eBioscience Ultracomp eBeads (San Diego, CA), and/or ArC Amine Reactive Compensation Beads (ThermoFisher Scientific). Cells were stained following the protocol outlined above for either surface staining or Live/Dead staining. Beads were stained following manufacturer protocol.

### Flow Cytometry

Samples were analyzed with the use of BD LSRII analyzers (Becton Dickinson, Franklin Lakes, NK) configured for 18-color analysis at the University of Pennsylvania Flow Cytometry and Cell Sorting Resource Laboratory or the Stanford Shared FACS Facility and a BD LSRFortessa analyzer (Becton Dickinson, Franklin Lakes, NK) configured for 18-color analysis at the PAVIR Flow Cytometry Core as described (25).

### Sorting

Samples were sorted on a BD FACSARIAIII (Franklin Lakes, NJ) in the VA Palo Alto Flow Cytometry Core and stored at -80**°**C as described (30).

### Nested PCR and Sequencing

Reverse transcription, nested amplification, barcoding, and library preparation were completed as described (30, 31). Next generation sequencing (NGS) was completed using Illumina MiSeq (San Diego, CA, USA) in the Stanford Functional Genomics Facility. Data were processed as described (31).

### Analysis of TCR Sequencing Data

Sequencing data were processed as described (26) and TCR clones were defined as follows:

1. If CDR3β is detected for a cell, then a clone will be defined as cells with identical CDR3β.
2. If CDR3α is detected and CDR3β is NOT detected for a cell, then a clone will be defined as cells with identical CDR3α (for either alpha chain detected, if there are two).
3. If neither CDR3α nor CDR3β are detected, the cell can be excluded from analysis.

### Batch Correction of Gene Expression Sequencing Data

Protein data were transformed using hyperbolic-arcsine transformation in Python (v.3.7.4), and RNA data using log-transformation as described (26).

### Data Analysis

Analysis and sorting were completed using FACSDiva software (BD, Franklin Lakes, NJ). Graphs were generated and statistics calculated in GraphPad Prism (San Diego, CA). Graphs include violin plots, pie charts, and bar plots. Flow cytometry data were analyzed in FlowJo version 10.7.1 (BD, Ashland, OR).

Sequencing data processed as described in sections 2.9 and 2.10 were imported into SeqGeq version 1.7.0 (BD, Ashland, OR). Data for all 20 genes were analyzed using Uniform Manifold Approximation and Projection (UMAP) dimensionality reduction version 3.1 (32). Clustering was completed using Seurat version 3.3 (33). Genes are denoted with (Ab) if detected by fluorescent staining, and with no extra notation if detected by RNA. We used VDJ Explorer version 2.0 in SeqGeq to identify TCR clones and the proportion of cells with each clone. For analysis of TCR, we defined expanded clones as clones appearing either in at least one cell at two or more time points or two cells in at least one time point. We defined rare clones as clones appearing in only one cell. This definition of clonality differs from that in the previously published papers using this data set (26, 27) in that the definition in those papers was based on percentages, not cell numbers.

### Statistics

Unpaired analysis of two groups was conducted through a Mann-Whitney test. One way comparison of 2 or more matched groups was computed using mixed-effects analysis with Tukey correction for multiple comparisons. Slope of change over time was computed with a simple linear regression. Two-tailed testing and alpha of 0.05 were used unless otherwise noted. Statistics for each comparison are listed in figure legends.

## Results

### CMV Seropositivity Influences CD4 T Cell Phenotype in Transplant Recipients

**Figure 1:**
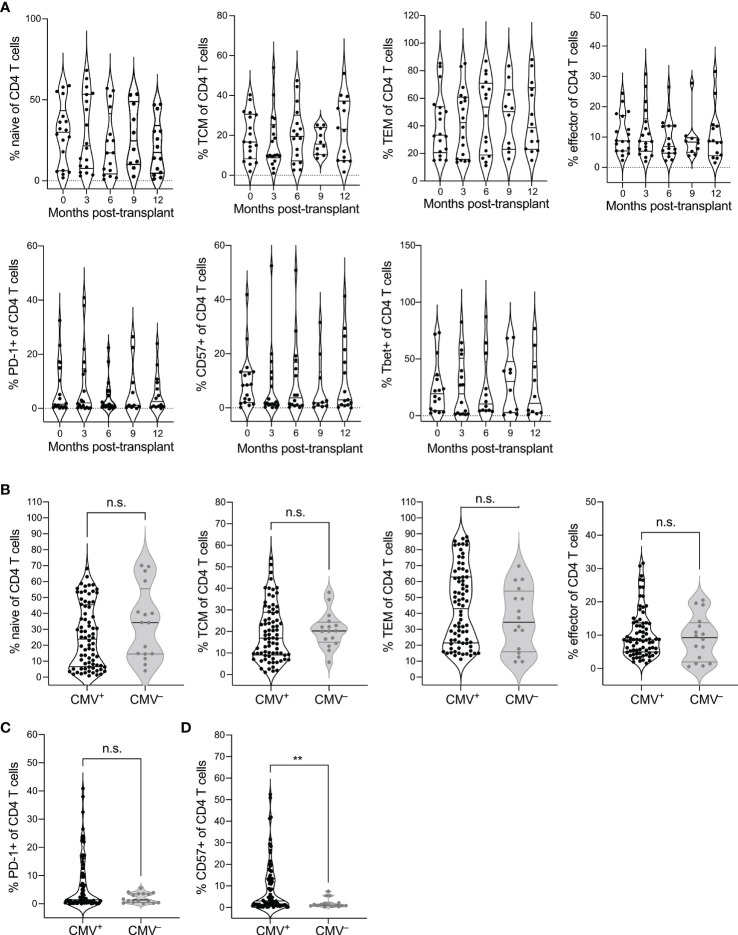
CMV seropositivity affects CD4 T cell differentiation. PBMC from CMV^+^ and CMV^–^ solid organ transplant recipients at time points pre- and 3, 6, 9, and 12 months post-transplant were evaluated by flow cytometry for differentiation states of CD4 T cells, gated as live CD3^+^CD8^–^CD4^+^CD14^–^CD16^–^CD19^–^. CD4 T cells were gated as naïve (CCR7^+^CD45RO^–^), central memory (TCM, CCR7^+^CD45RO^+^), effector memory (TEM, CCR7^–^CD45RO^+^), and effector (CCR7^–^CD45RO^–^), or PD-1^+^, CD57^+^, or T-bet^+^. **(A)** Frequencies of (top) naïve, TCM, TEM, and effector CD4 T cells and (bottom) PD-1^+^, CD57^+^, and T-bet^+^ cells at individual time points from pre- to a year post-transplant at three month intervals. Population frequencies were compared between CMV^+^ and CMV^–^ subjects for **(B)** naïve vs memory state, **(C)** PD-1^+^ cells, and **(D)** CD57^+^ cells across all time points. Violin plots depict density, and individual points represent individual samples. Statistics computed with **(A)** mixed-effects model with Tukey correction for multiple comparisons or **(B–D)** Mann-Whitney test. n.s. = not significant ** = p < 0.01. Data set previously published for analysis of CD8 T cells (25). n= 6 (CMV^–^), 16-20 (CMV^+^).

To identify the impact of CMV seropositivity and transplantation on CD4 T cell immunity, we analyzed CD4-gated PBMC from CMV^+^ and CMV^–^ recipients of heart or kidney transplant by flow cytometry (**Supplementary Table 1**). Using CCR7 and CD45RO expression to define differentiation states (**Supplementary Figure 1**), we first addressed whether CD4 T cell differentiation states changed post-transplant in CMV^+^ recipients by comparing phenotypes pre-transplant and at 3-month intervals during the first-year post-transplant (**Figure 1A**). We observed no statistical changes over time in frequency of naive (CCR7^+^CD45RO^–^), central memory (TCM, CCR7^+^CD45RO^+^), effector memory (TEM, CCR7^–^CD45RO^+^), or effector (CCR7^–^CD45RO^–^) T cells (**Figure 1A**, top row, top, p > 0.06). However, there was a trend of decrease in naïve (month 3-12 p=0.066, month 9-12 p = 0.064) and central memory T cells (month 3-12 p=0.069, **Figure 1A**). We then analyzed expression of PD-1 as a measure of follicular helper T cell phenotype and CD57 as a measure of immune aging. CMV is known to drive Th1 differentiation and aging of CD4 T cells in immunocompetent subjects (34). We also included analysis of T-bet expression, a transcription factor required for Th1 differentiation. Immune aging and CMV are each associated with increased T-bet expression (35). There were no statistical changes in proportion of CD4 T cells expressing PD-1, CD57, or T-bet (**Figure 1A**, bottom row, p > 0.05). For all phenotypes depicted in **Figure 1A**, the slope of change after transplant was not significantly non-zero (p>0.23). Thus, CD4 T cell differentiation states were largely stable in the first year after transplantation in CMV^+^ recipients.

We next wanted to determine whether there is a difference in CD4 T cell differentiation between CMV^+^ and CMV^–^ transplant recipients. Because of the lack of trend over time, we pooled data from all time points for this comparison. We found trends towards decreased naive (p=0.14), and increased TEM populations (p=0.25) in the CMV^+^ recipients (**Figure 1B**). There was no statistical difference in TCM (p=0.42) or effector (p=0.66) T cells between CMV^+^ and CMV^–^ recipients (**Figure 1B**). CMV^+^ recipients had a trend towards elevated percentage PD-1^+^ (**Figure 1C**, p=0.25) and a statistically increased percentage that were CD57^+^ (**Figure 1D**, p=0.0069) compared with CMV^–^ transplant recipients. Thus, CMV is associated with increased differentiation of CD4 T cells in transplant recipients, including elevated immune aging and potentially elevated Tfh population, but there are no changes associated with CMV in the first year after transplant.

### CMV-Responsive CD4 T Cells Comprise a Variety of Differentiation States in Transplant Recipients

**Figure 2:**
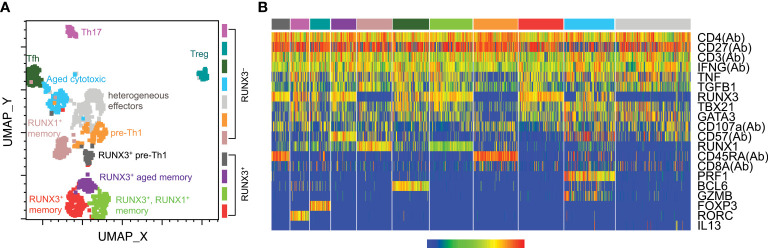
CMV-responsive CD4 T cell cluster classification. CD4 T cells from six transplant recipients were stimulated with CMV IE-1 peptide for 5 hours and IFNγ-positive cells sorted for single-cell targeted PCR and sequencing as described (26, 31). Data for 20 detected genes were batch-corrected as described (26). Cells were gated as CD4^+^ in SeqGeq and concatenated for analysis. Seurat was run in SeqGeq for dimensionality reduction and clustering. **(A)** UMAP dimensionality reduction color-coded by the Seurat clusters, with cluster names as defined in part **(B)** found on the plot. **(B)** Seurat generated heatmap displaying gene expression of each cluster. Parameters with (Ab) were detected at the protein level, and the others were detected at the RNA level. Classifications were assigned based on gene expression patterns. n=6 subjects.

Phenotypic analysis indicated an impact of CMV on total CD4 T cells, but did not specifically analyze the subset responding to CMV stimulation. In order to characterize CMV-responsive CD4 T cells, we used targeted single cell sequencing to detect TCR and expression of 20 genes (31). We isolated single IFNγ^+^ CMV-responsive T cells from six solid organ transplant recipients (**Supplementary Table 2**) at pre-, 3, and 12 months post-transplant using IFNγ capture after 5 hours of CMV IE-1 peptide stimulation as described (26, 27). We selected subjects to represent both donor CMV^+^ and CMV^–^ subjects, and both lymphodepleting (rATG) and non-lymphodepleting (steroid with or without αIL2R) induction therapies. We measured TCR and gene expression through nested PCR and single cell sequencing. To analyze all 20 parameters together, we used UMAP dimensionality reduction and Seurat clustering (**Figure 2A**). Seurat identified 11 clusters (**Figure 2A**), with a heatmap to classify gene expression (**Figure 2B**).

The clusters represented a variety of CD4 T cell differentiation states. The majority of clusters expressed T-bet, consistent with Th1 differentiation states, and with the IFNγ production by this population. Other Th populations were defined based on FOXP3 (Treg), *RORC* (Th17), and BCL6 (Tfh). The other eight clusters all show characteristics of Th1 differentiation, and are named based on the characteristics that subdivide them within the Th1 state (**Figure 2A**). The four clusters in the lower half of the plot are RUNX3^+^, which is associated with Th1 differentiation (36, 37) and cytotoxicity (24, 38). These clusters all represented memory cells based on CD27 expression, and were further subdivided as aged (CD57^+^) and RUNX1^+^ or ^–^. The upper half were RUNX3^–^ cells, including memory, aged, and effector cells, as well as the additional aforementioned Th states. The CD57^+^RUNX3^–^ cluster also contains cytotoxic cells, in this case defined by high levels of granzyme B (GZMB) and perforin 1 (PRF1). Two clusters had features of both undifferentiated and Th1 cells, with co-expression of CD45RA and CD27 and low expression of T-bet, RUNX1 and TNF. We refer to these clusters as “pre-Th1,” reflecting that the Th1 cytokine IFNγ and their relative lack of other Th1 phenotypes suggest they are an early Th1 population. The two pre-Th1 clusters, much like the six Th1 clusters, can be subdivided into RUNX3^+^ and RUNX3^–^, suggesting differential cytotoxicity between the two populations. Thus, IFNγ-producing CMV-responsive CD4 T cells comprise a wide range of differentiation states, but predominantly Th1 and cytotoxic.

### CD4 T Cells Are Phenotypically Stable Over the First Year Post-Transplant

**Figure 3:**
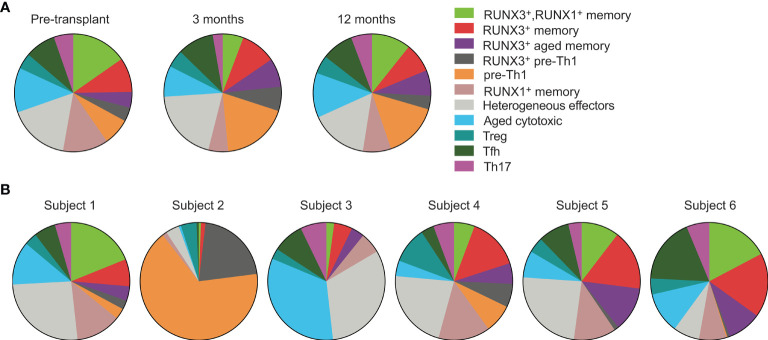
CMV-responsive CD4 T cells have similar phenotypes across subjects and time points. Cell clusters were defined in **Figure 2**. **(A)** CD4^+^ gated cells from all six subjects were concatenated for analysis by **(A)** time point and **(B)** individual subject. Data were analyzed **(A)** pre-transplant, 3 months, and 12 months post-transplant on 392 cells, 330 cells, and 400 cells, respectively, and **(B)** across subjects 1-6 on 201, 161, 239, 140, 163 and 318 cells, respectively. n=6 subjects.

We next analyzed how the phenotypes vary over time and between subjects. We began by evaluating cluster frequencies at each time point including all subjects (**Figure 3A**). With the exception of the RUNX3^–^ pre-Th1 population which increased slightly from pre- to 3 months post-transplant, we found that the majority of clusters appeared at similar frequencies at each time point, in particular the populations of effector, Treg, memory and aged cytotoxic cells. We then compared the populations for each individual subject across time points (**Figure 3B**). Each subject’s CD4 T cells included at least 9 of the 11 clusters. Specifically, subjects 1, 4, and 6 had all clusters represented, subject 2 lacked the Th17 cluster, subject 3 lacked both pre-Th1 clusters, and subject 5 lacked the RUNX3^–^ pre-Th1 cluster. These findings suggest consistent CD4 T cell differentiation in transplant recipients, particularly when excluding the pre-Th1 population. However, the frequencies of populations varied. In particular, the RUNX3^–^ pre-Th1 population was dominant in subject 2, in a striking difference from all other subjects (**Figure 3B**). Subject 3 also displayed a higher frequency of aged cytotoxic cells compared to the other subjects, while subjects 1, 4, 5, and 6 had largely consistent populations and frequencies (**Figure 3B**).

Because the above analyses did not account for heterogeneity between subjects and over time, we also analyzed the clusters in each subject at each time point (**Supplementary Figure 2**). Subjects 2, 4, and 6 had stable phenotypes through the first year post-transplant. Subject 2 had predominantly RUNX3^–^ pre-Th1 cells whereas subjects 4 and 6 had diverse CD4 T cell phenotypes. The cluster frequencies of subjects 1, 3, and 5 changed substantially from pre- to post-transplant. Subjects 1 and 3 decreased in phenotypic diversity by a year after transplant, from nine or eleven different clusters to four or five clusters respectively. In contrast, subject 5 had increased immune cell diversity from four to nine clusters by three months post-transplant. The predominant phenotypes in samples with fewer than nine clusters were the heterogeneous effector and aged cytotoxic clusters. From 3 to 12 months post-transplant, cluster frequencies were stable across all subjects.

### Clonally Expanded CMV-Responsive CD4 T Cells Are Predominantly Aged Cytotoxic

**Figure 4:**
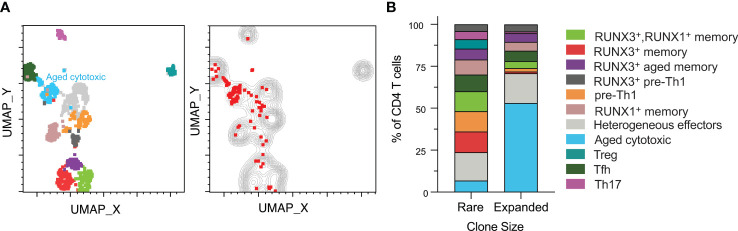
Clonally expanded CD4 T cells have a distinct aged cytotoxic phenotype. CD4^+^ T cell data from all six subjects and time points were gated for TCR analysis. **(A)**, left UMAP plot from **Figure 2** of all samples depicted with aged cytotoxic cluster labeled. **(A)**, right Rare clones are displayed as a gray contour plot. Red dots represent expanded clones as defined by the presence of one cell of a clone at different time points or two or more cells at the same time point. All cells from all subjects are depicted in either the gray or the red. **(B)** Rare and expanded clones were organized into a stacked bar graph of cluster frequencies. n=6 subjects.

The heterogenous phenotypes observed led us to investigate whether clonally expanded CMV-responsive CD4 T cells were represented by specific phenotypes. We analyzed clonal expansion in data concatenated for all subjects and time points (**Figure 4A**). Specifically, we defined expanded clones as TCR clones that occurred in at least two sequenced cells in one subject. This definition included either two or more cells at the same time point or at least one cell present at more than one time point (red dots on the plot). The rationale behind this definition was that a clone that is represented in only one cell is of indeterminate size, but a clone represented in at least two cells in our data is clonally expanded, though exact quantitation would require higher numbers of cells. We defined those clones only occurring in one cell as rare (gray contour). All CMV-responsive CD4 T cells from all six subjects are depicted in either the gray contour or red dots. Rare TCR clones were approximately 9 times more abundant than the clonally expanded population, and were represented all 11 clusters (**Figure 4A**). In contrast, the expanded clones were predominantly in the aged cytotoxic and effector clusters. Expanded clones were detected in all subjects except subject 4, with only subjects 1, 3, and 6 ever having more than 2% clonally expanded of CMV-responsive CD4 T cells. The largest expanded populations were detected in subjects 1 and 3 pre-transplant (8.2% and 10.9% of CMV-responsive CD4 T cells respectively), and in subject 6 at three months post-transplant (8.3%). Each of these subjects had lower frequencies of expanded cells at the other two time points (0.6-2.6%). Thus, expanded clones represented a small fraction of CMV-responsive T cells, but one with a distinct phenotype.

The rare clones were well-distributed by phenotype with the largest cluster being heterogeneous effectors at approximately 17% (**Figure 4B**). In contrast, the aged cytotoxic cluster comprised over 50% of the total expanded population. Heterogeneous effectors made up the second highest percentage of expanded clones, which was similar in frequency to their non-expanded counterparts. When comparing across subjects, cluster distributions of expanded and rare clones varied. Aged cytotoxic and heterogeneous effector were the dominant clusters in the three subjects with significant clonal expansion (1, 3, and 6), representing 56-100% of clonally expanded cells at the time point with the largest clonally expanded population. The RUNX3^+^ aged memory and RUNX3^+^ pre-Th1 were at similar frequencies in the expanded and rare populations. The one cluster absent from the expanded clones was the Treg cluster, and all other clusters were represented at lower frequencies than in the rare clones. Overall, cytotoxicity and aging appear to drive CMV-responsive CD4 T cell differentiation in the context of clonal expansion.

## Discussion

In this study we hypothesized that CD4 T cell differentiation and function were impacted by transplantation and the associated therapies, consistent with memory inflation and immune aging of CD8 T cells after transplantation. To address this hypothesis, we used flow cytometry and targeted single cell TCR and RNA sequencing in longitudinal samples of CD4 T cells from pre- to one year post-transplant. We found that CD4 T cells were phenotypically more aged in CMV^+^ than CMV^–^ transplant recipients, and that there was a non-statistical trend towards reduction of naïve T cells as a proportion of CD4 T cells in the first year after transplant. CMV-responsive CD4 T cells were composed of diverse phenotypes in CMV^+^ transplant recipients, which were maintained from pre- to one year post-transplant. The phenotypes present varied from patient to patient, with the greatest discrepancy in the populations that we termed pre-Th1. Clonally expanded T cells were highly enriched for an aged cytotoxic phenotype, whereas rare clones represented all phenotypes. Overall, CMV-responsive CD4 T cells maintained phenotype and functionality after transplantation, and the clonally expanded subset appeared to represent a population protective against CMV.

While the majority of studies of CMV-responsive T cells have focused on CD8 T cells, CD4 T cells play an important role as well. Low levels of CD4 T cells responsive to stimulation with CMV antigen correlate with symptomatic CMV disease after kidney (8) or hematopoietic stem cell (6, 10) transplant. Studies in healthy children also show that the development of the CD4 T cell response to CMV is required to eliminate viral shedding in the urine (7). Thus, CD4 T cell-mediated immunity is crucial to the control of CMV replication, and important to understand in the context of transplantation. Furthermore, as the majority of studies of CMV-specific T cell repertoire have focused on CD8 T cells, so this study fills several important gaps in the knowledge of CMV-responsive CD4 T cells: longitudinal analysis post-transplant, TCR repertoire, and concomitant analysis of phenotype and TCR.

An important factor in understanding CD4 T cell immunity to CMV is the interplay of CMV replication and T cell responses. The subjects in our cohort had no detected episodes of CMV viremia nor clinical signs of CMV disease, though asymptomatic self-resolving viremia would not have been detected. The subjects included in the sequencing analysis were also all CMV^+^, meaning they were at intermediate risk of CMV disease, and had memory T cells able to contribute to control of CMV. Thus, these subjects had known latent CMV infection during the study period, with the possibility of undetected transient viremia. Pre-emptive monitoring for CMV has demonstrated that transient self-resolving viremia is common in transplant recipients (39), and transplant recipients have elevated CMV-specific immunity relative to healthy volunteers, suggesting a response to undetected viremia (40). Our prior work has suggested that low level viral replication can drive memory inflation of CD8 T cells after transplantation, but that CD4 T cell memory inflation does not occur in the same context (11, 26, 27). CD4 T cell memory inflation has been observed in humans (41), leading to the question of how CD4 T cell differentiation may be altered in this context that promotes CD8 but not CD4 inflation.

Our data identifying memory and cytotoxic phenotypes in CMV-responsive CD4 T cells extend previous studies identifying a highly differentiated effector memory phenotype (41). The enrichment of the aged cytotoxic population in clonally expanded IFNγ+ cells is consistent with a previous study of the phenotype and function of CD4 T cells binding CMV-HLA tetramers. This study found that these CMV-specific CD4 T cells were cytotoxic, and that the cytotoxicity increased with age (42). Our data identifying an aged phenotype in the cytotoxic population further suggest enhanced aging. CMV-specific CD4 T cells have also been shown to be cytotoxic in the mouse model murine CMV (43). Our RUNX3 data provide evidence of CD4 T cell cytotoxicity in transplant recipients, as this gene has been shown to promote Th1 differentiation and cytotoxicity in CD4 T cells (23, 24). Of note, the majority of clusters in our data did not express granzyme or perforin mRNA. Analysis of granzyme expression in our data was limited to granzyme B, so we cannot eliminate the possibility that these CD4 T cells expressed other granzymes. However, a portion of cells in all clusters, including all four RUNX3^+^ clusters, expressed CD107a, which is a marker of degranulation (44). Additionally, CD8 T cells typically have higher levels of granzyme and perforin than CD4 T cells, even in memory populations that exhibit comparable killing activity (45, 46). Thus, in CD4 T cells, CD107a expression and direct killing assays are more accurate measures of cytotoxicity than granzyme and perforin expression. Overall, we have found IFNγ producing CMV-responsive CD4 T cells to be phenotypically cytotoxic, with a significant fraction that are aged. Further, our study suggests that clonal expansion drives expansion of aged cytotoxic CD4 T cells at the expense of populations that are not cytotoxic, indicating that for clonal expansion, cytotoxicity and aging may be more important drivers of differentiation than the Th1 state. These findings are consistent with this population participating in the response protective against CMV.

In addition to the cytotoxic phenotype, our data identified several other differentiation states in CMV-responsive CD4 T cells. One such population, Treg, is of potential interest because of evidence that CMV-specific Treg may attenuate the vascular damage caused by CMV-specific CD8 T cells in the elderly (47). Thus, this population may protect patients by regulating other T cell populations. Another population that stood out was Tfh, a subset that promotes B cell responses. Tfh interact with B cells in specific tissue sites and not in the blood. Therefore, the population in our study reflects a circulating Tfh population that shares phenotype and function with the cells promoting B cell activity in tissues (48). Tfh are typically more important to the primary than secondary response to CMV, though post-transplant CMV reactivation can drive expansion of this population (49). A final population of interest are Th17, a highly inflammatory population commonly associated with autoimmune disease (50). In a mouse model of CMV and kidney transplant, IL6 blockade reduced infiltration of Th17 into the allograft, as well as allograft injury (51). While the numbers of cells were low enough to limit quantitative analysis, we detected six clones that were represented by both Th1 and Tfh phenotypes, one represented by Th1 and Th17 phenotypes, and one exclusively represented by Th17 phenotype, though the remaining 26 were represented exclusively by Th1 phenotypes. Thus, multiple populations of CD4 T cells identified in this study could impact transplant health in different ways.

An important consideration for interpretation of the Th subsets detected is that by sorting IFNγ^+^ cells after CMV stimulation, we enriched for a Th1 phenotype. Therefore, we would not expect to see a dominant Th2, Th9, Th17, Tfh, or Treg response specifically. This data set provides significant insight into the Th1 arm of the CMV response, but further study will be important to understand the role of other Th subtypes in response to CMV. Towards this end, we would recommend follow up studies isolating CMV-responsive CD4 T cells in a manner not skewed towards Th1, for example with staining for CD154 after stimulation or use of MHC II:peptide multimer reagents.

The populations in our study with the most variation from subject to subject were the clusters we termed pre-Th1. We have not found a direct analog for these clusters in the literature, but defined them based on shared IFNγ with Th1, and the relative lack of expected T-bet (52). Thus we have inferred, but not proven, that they are an early Th1 population. The high degree of variability of this population across subjects relative to other populations suggests stochasticity of the initial stages of CMV-responsive CD4 T cell differentiation, and stability of phenotype once the cells have reached a more differentiated state.

One factor that is important to contextualize these findings is the limited patient cohort. The analysis was in six individuals, who were selected to represent both lymphodepleting (rATG) and non-lymphodepleting induction therapies (**Supplementary Table 2**). All six received similar standard of care three-drug immunosuppression, but no further restrictions were made on enrollment. The difference in induction does not appear to affect the CD4 T cell phenotypes, with those subjects with rATG induction (3, 4, and 6) having CD4 T cells with stable phenotypes post-transplant. Three subjects had changes in phenotype from pre- to 3 months post-transplant, but two of these (subjects 1 and 5) received non-lymphodepleting induction, so the change from pre- to post-transplant must be associated with a different peri-transplant event. The differences observed between subjects are also not explained by donor CMV serostatus, as those with changes from pre- to post-transplant include both CMV^+^ and CMV^–^ donors. Further study will be needed to determine the factors affecting whether or not the CD4 T cell phenotype changes from pre- to post-transplant. Regardless, the phenotypes are largely stable in the post-transplant period observed, indicating that analysis at three months post-transplant can be expected to reflect T cell populations nine months later.

When comparing these findings to previously published research on CMV-responsive CD4 T cells, it is important to consider distinctions in study design. Specifically, CMV-responsive CD4 T cells have been more often identified with the use of CMV lysate or phosphoprotein 65 (pp65) stimulation (7, 8, 10, 19), and we stimulated with peptide from IE-1, which is a dominant antigen for CD8 T cells, and less so for CD4 T cells (29). While a population responding to lysate would include the population analyzed in our study, the pp65-responding population is likely distinct. In these studies, CMV-responsive CD4 T cells produced IFNγ and other cytokines, and exhibited cytotoxicity against infected cells (7, 8, 19). These phenotypes are consistent with our observations, but further study of CD4 T cell responses to pp65 will be important to address whether the clonality observed still applies with this immunodominant stimulus. Further, expansion of CMV-responsive CD4 T cells over time has been specifically detected in the context of stimulation with lysate or a library of peptides across all CMV open reading frames (41, 53), so IE-1 may or may not drive expansion of this population. In our analysis of these subjects so far, we have not detected expansion of CD4 T cells in response to either IE-1 or pp65. We therefore recommend further study of CD4 responses to CMV lysate to determine the impact of expansion on clonality.

While memory inflation is typically considered a feature of CD8, not CD4, T cell responses to CMV (54), there is evidence of expansion of the CMV-responsive CD4 T cell population over time in both aging humans and mice (41, 53). We did not detect memory inflation of CD4 T cells in our cohort despite the CD8 memory inflation observed (27); this finding suggests different time courses of population expansion in the two T cell types. Further study of pp65-responsive T cells will be important to clarify the time frame of CD4 T cell expansion.

One finding that merits further discussion was the distinct CD4 T cell phenotype in subject two, with dominance of the naïve subset. This was unexpected given that the isolated population was CMV-responsive T cells. Analysis of CMV-responsive CD8 T cells from the same individual indicated that the CD8 population was clonally expanded and highly differentiated (26, 27), making this CD4 T cell finding quite striking in contrast. While limited to one subject, this analysis demonstrates that concurrent analysis of CD4 and CD8 T cell CMV responses will be important to understanding the global immune response to CMV.

Overall, our study provides an in-depth analysis of CD4 T cell immunity to CMV during the first year after solid organ transplantation. Our most striking finding was phenotypic stability both across and within subjects during that period, including in recipients who received lymphodepleting induction. Further, we found that IFNγ-expressing CD4 T cells that clonally expanded in response to CMV had an aged and cytotoxic phenotype, consistent with the phenotypes we had previously observed in CD8 responses. There are therefore overlaps and differences between these two T cell populations, indicating that further study of the CD4 response will be important to gain the full picture of T cell immunity to CMV in transplant recipients. Furthermore, we identified a heterogeneous CD4 T helper cell response to CMV that could be protective against CMV-associated pathology (Treg), could enhance pathology (Th17), or could be associated with reactivation events (Tfh). Incorporating the study of these populations in further studies will also be important to understand the relative risks associated with these cells. This study provides detailed information about CD4 T cell responses to CMV after transplantation that will be important to design follow up studies to address direct impact of these populations on transplant recipients.

## Data Availability Statement

The datasets analyzed in this study can be found in the Sequence Read Archive under accession number PRJNA75237 (https://www.ncbi.nlm.nih.gov/bioproject/PRJNA752378).

## Ethics Statement

The studies involving human participants were reviewed and approved by IRBs at the University of Pennsylvania (protocol number 817637), the VA Palo Alto Health Care System and Stanford University (protocol number 38882). The patients/participants provided their written informed consent to participate in this study.

## Author Contributions

LEH completed experiments. LEH, AAA, and SS completed analyses of the data. LEH and JSM contributed to experimental design. KBM oversaw subject enrollment and sample procurement at the University of Pennsylvania. LEH, AAA, and JSM wrote the manuscript. All authors contributed to the article and approved the submitted version.

## Funding

This work was supported by awards to JSM from the American Heart Association (13IRG13640042) and the Veterans Administration (1I01CX001971) and LEH from the Stanford Translational Research and Applied Medicine Program. LEH received support from Enduring Hearts and the American Heart Association (17POST33660597) and the National Institutes of Health [T32 AI07290; K01 1K01DK123196]. AAA received support from the National Institutes of Health (Stanford PreRenal Initiative 1R25DK122957-01A1).

## Conflict of Interest

JSM has a family member who is employed by and has an equity interest in Genentech/Roche. No patents have been filed pertaining to the results presented in this paper.

The remaining authors declare that the research was conducted in the absence of any commercial or financial relationships that could be construed as a potential conflict of interest.

## Publisher’s Note

All claims expressed in this article are solely those of the authors and do not necessarily represent those of their affiliated organizations, or those of the publisher, the editors and the reviewers. Any product that may be evaluated in this article, or claim that may be made by its manufacturer, is not guaranteed or endorsed by the publisher.

## Abbreviations

BCL6, B-cell lymphoma 6; CCR7, C-C chemokine receptor; CMV, Cytomegalovirus; CMV^+^, CMV seropositive; CMV^–^, CMV seronegative; EDTA, Ethylenediaminetetraacetic acid; Tfh, Follicular helper T cell; GATA3, GATA Binding Protein 3; GZMB, Granzyme B; IE-1, Immediate early-1; IFNγ, Interferon gamma; NGS, Next generation sequencing; PRF1, Perforin 1; PBMC, Peripheral blood mononuclear cells; pp65, phosphoprotein 65; PE, Phycoerythrin; RORγt, Retinoid orphan receptor gamma t; T-bet, T-box expressed in T cells; TCR, T cell receptor; Th1, T-helper 1; TNFα, Tumor necrosis factor α; Th2, T-helper 2; UMAP, Uniform Manifold Approximation and Projection.
