# Supplementary material for: CMV-Responsive CD4 T Cells Have a Stable Cytotoxic Phenotype Over the First Year Post-Transplant in Patients Without Evidence of CMV Viremia
Source: Front Immunol. 2022 Jun 28;13:904705. doi: 10.3389/fimmu.2022.904705 (PMC9275561; doi:10.3389/fimmu.2022.904705)
Supplement: Supplementary file 1 [file DataSheet_1.docx]

**Supplementary Figures and Tables**

**Supplementary Figure 1: Gating scheme.** Gating schematic used to identify live singlets (top row), and CD4 T cell differentiation states (bottom row) in Figure 1.

**Supplementary Figure 2: Phenotypic variation across subjects.** Cell clusters were classified as described in Figure 2. Each row is an individual patient, and the columns are pre-transplant, 3 months, and 12 months post-transplant. n=6 subjects

| Recipient CMV serostatus | # of subjects | % male | Age | % Donor CMV^+^ | % rATG | % αIL-2R |
| --- | --- | --- | --- | --- | --- | --- |
| – | 6 | 100% | 62 (52.5-69.25) | 33% | 83% | 17% |
| + | 20 | 75% | 58 (49.25-65.25) | 35% | 55% | 35% |

**Supplementary Table 1: Clinical and demographic details on subjects in Figure 1.** Data depicted represent the 26 subjects analyzed in figure 1. rATG = rabbit anti-thymocyte globulin, αIL-2R = anti-interleukin-2 receptor. Table summarizes data for CMV^+^ and CMV^–^ recipients separately. % indicates percentage of subjects with that condition. Table modified from previously published paper (1).

| **#** | **Transplant** | **Sex** | **Age** | **Donor CMV** | **Recipient CMV** | **Induction therapy** |
| --- | --- | --- | --- | --- | --- | --- |
| 1 | Heart | F | 66 | – | + | αIL-2R, steroid |
| 2 | Heart | M | 58 | – | + | Steroid |
| 3 | Kidney | M | 68 | + | + | rATG, steroid |
| 4 | Kidney | M | 50 | – | + | rATG, steroid |
| 5 | Heart | M | 58 | + | + | αIL-2R, steroid |
| 6 | Kidney | M | 41 | + | + | rATG, steroid |

**Supplementary Table 2: Clinical and demographic details on subjects in Figures 2-4.** Data depicted represent the six subjects analyzed in figures 2-4. rATG = rabbit anti-thymocyte globulin, αIL-2R = anti-interleukin-2 receptor. Table modified from previously published papers (2, 3).

**References**

1. Higdon LE, Gustafson CE, Ji X, Sahoo MK, Pinsky BA, Margulies KB, et al. Association of Premature Immune Aging and Cytomegalovirus After Solid Organ Transplant. Front Immunol. 2021;12.

2. Higdon LE, Schaffert S, Cohen RH, Montez-Rath ME, Lucia M, Saligrama N, et al. Functional Consequences of Memory Inflation after Solid Organ Transplantation. The Journal of Immunology. 2021;207(8):2086-95.

3. Higdon LE, Schaffert S, Huang H, Montez-Rath ME, Lucia M, Jha A, et al. Evolution of Cytomegalovirus-Responsive T Cell Clonality following Solid Organ Transplantation. The Journal of Immunology. 2021;207(8):2077-85.
